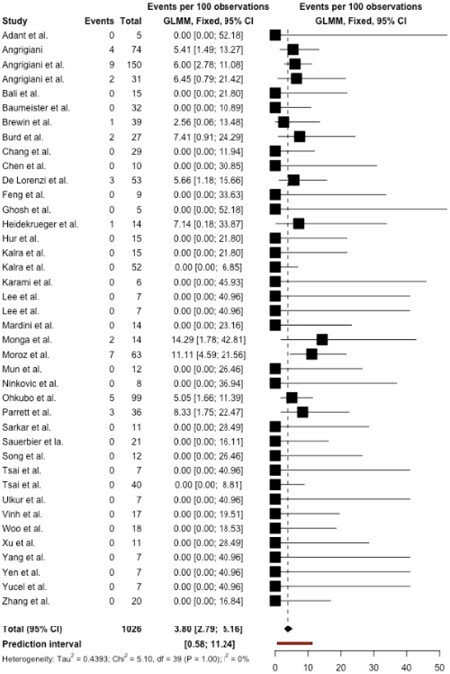# 82 Free Flap Failure and Contracture Recurrence in Delayed Burn Reconstruction: A Systematic Review and Meta-analysis

**DOI:** 10.1093/jbcr/irae036.081

**Published:** 2024-04-17

**Authors:** Jose Antonio Arellano, Hilary Liu, Mario Alessandri-Bonetti, Julia Kasmirski, Guy M Stofman, Francesco Egro

**Affiliations:** University of Pittsburgh Medical Center, Pittsburgh, PA; University of Pittsburgh Medical Center, Pittsburgh, PA; University of Pittsburgh Medical Center, Pittsburgh, PA; University of Pittsburgh Medical Center, Pittsburgh, PA; University of Pittsburgh Medical Center, Pittsburgh, PA; University of Pittsburgh Medical Center, Pittsburgh, PA

## Abstract

**Introduction:**

Free tissue transfer is often considered a last resort in burn reconstruction due to its complexity and associated risks. Free flap and contracture recurrence outcomes in delayed burn reconstruction remain unclear as reported in the literature, and a comprehensive review on free flap outcomes in delayed burn reconstruction is currently lacking. The study aims to evaluate the available evidence on the failure and contracture recurrence rates in free flap delayed burn reconstruction.

**Methods:**

A systematic review and meta-analysis was conducted and reported according to PRISMA guidelines. The protocol was registered on PROSPERO (CRD42023404478). PubMed, Embase, Web of Science, and Cochrane Library databases were queried. The results were limited to English-language literature with extractable data relating to microsurgical delayed burn reconstruction only. The measured outcomes were free flap loss and contracture recurrence rate.

**Results:**

Of the 1262 retrieved articles, 40 qualified for inclusion, reporting on 1026 free flaps performed in 928 patients (50.3% male, 49.7% female). The mean age was 29.25 years [95% CI: 24.63, 33.88]. Delayed burn reconstruction was performed at an average of 94.68 months [95% CI: -9.34, 198.70] after initial injury, with a follow-up period of 23.02 months [95% CI: 4.46, 41.58]. Total flap loss (TFL) rate was 3.80% [95% CI: 2.79, 5.16] (Figure 1) and partial flap loss (PFL) rate was 5.95% [95% CI: 4.65, 7.57]. Interestingly, burn contracture recurrence rate was 0.62% [95% CI: 0.20, 1.90] (Figure 2).

**Conclusions:**

This systematic review provides a comprehensive evaluation of the free flap outcomes in delayed burn reconstruction. The flap loss rate was relatively low, given the complexity of the procedure and potential risks. Furthermore, burn contracture rate was found to be extremely low. This study demonstrates that free flaps are a safe and effective option for delayed burn reconstruction.

**Applicability of Research to Practice:**

The findings of this systematic review suggest that free flap procedures are a viable and relatively safe option for delayed burn reconstruction, with low rates of flap loss and burn contracture recurrence, providing valuable insights for researchers exploring reconstructive options in burn injury cases.